# Future pharmacists and climate action: a qualitative study of students’ views on environmental sustainability in education and practice

**DOI:** 10.1007/s11096-025-02053-4

**Published:** 2025-11-22

**Authors:** Kingston Rajiah, Annabelle McArthur

**Affiliations:** School of Pharmacy and Pharmaceutical Sciences, Ulster University, Coleraine, BTS2 1SA UK

**Keywords:** Climate change mitigation, Curriculum, Environmental Sustainability, Pharmacists, Students, pharmacy

## Abstract

**Introduction:**

Awareness of planetary health has grown across healthcare professions, and the pharmacy sector, responsible for the lifecycle of medicines from production to disposal, plays a significant role in both perpetuating and mitigating environmental harm. However, pharmacy education omits environmental sustainability as a structured component.

**Aim:**

To explore pharmacy students’ perceptions of environmental sustainability within their education and future professional roles, focusing on their awareness, attitudes, and perceived barriers or enablers to integrating sustainability in pharmacy education and practice.

**Method:**

A qualitative study was conducted at a university in Northern Ireland, utilising semi-structured interviews with pharmacy students across all four years of their studies. Purposive sampling was used. Interviews were transcribed verbatim and analysed inductively using Braun and Clarke’s six-phase approach to thematic analysis.

**Results:**

Sixteen students were interviewed, with data saturation reached at the 14th interview and confirmed through researcher consensus and transcript review. Six themes emerged (mean interview length = 45 ± 10 min): (1) Sustainability as an overlooked area – limited or fragmented curricular coverage; (2) Narrow understanding of waste – awareness focused on disposal and packaging, with little recognition of wider pharmaceutical impacts; (3) Pharmacy as both waste generator and sustainability site – examples included medicines returns, digitalisation, and hospital “green teams”; (4) Integrating sustainability into education – preference for interactive, experiential learning (e.g., guest speakers, placements); (5) Barriers to change – patient resistance, behavioural inertia, and competing curricular demands; (6) Policy, incentives, and leadership as enablers – systemic and financial support deemed essential for sustainable practice.

**Conclusion:**

Pharmacy students recognise the importance of environmental sustainability but perceive significant gaps in both education and practice. While they value opportunities for experiential learning and see potential for pharmacy to contribute positively, systemic barriers and limited curricular integration hinder progress. Embedding sustainability into pharmacy education, supported by leadership, policy, and incentives, will be critical to preparing future pharmacists as both healthcare providers and environmental stewards.

**Supplementary Information:**

The online version contains supplementary material available at 10.1007/s11096-025-02053-4.

## Impact statements

- Embedding sustainability into pharmacy curricula can better prepare graduates to act as environmental stewards while maintaining high-quality patient care.
- There are opportunities for pharmacies to reduce their environmental footprint through medicine disposal schemes, digitalisation, and sustainable packaging.
- Systemic support, including leadership, incentives, and policy alignment, is needed to enable sustainable practices across the profession.
- Incorporating the student voice into curriculum reform ensures future pharmacists are equipped with relevant knowledge, skills, and motivation to address healthcare’s environmental challenges.

## Introduction

Environmentally sustainable pharmacy practice refers to the application of environmentally responsible principles across the lifecycle of medicines from procurement and prescribing to dispensing, use, and disposal to minimise the environmental footprint of pharmaceutical activities while maintaining patient safety and therapeutic effectiveness [1]. Awareness of planetary health has grown across healthcare professions, and the pharmacy sector, responsible for the lifecycle of medicines from production to disposal, plays a significant role in both perpetuating and mitigating environmental harm [2–4]. Pharmaceutical products contribute notably to healthcare’s carbon footprint and environmental pollution [5]. Metered dose inhalers (MDIs), for example, account for almost 1% of the healthcare sector’s greenhouse gas emissions due to propellant use [6]. Beyond this, the manufacture of active pharmaceutical ingredients (APIs) is highly energy intensive, involving significant solvent use and chemical pollution, while the improper disposal of pharmaceuticals contributes to antibiotic resistance and ecological disruption [7]. Pharmacists, as medicine experts and frontline healthcare providers, therefore, bear a pivotal responsibility in promoting the sustainable use and management of medicines. Despite this clear mandate, studies reveal that pharmacy education often omits environmental sustainability as a structured component. Across international contexts, pharmacy students report low familiarity with sustainable pharmacy practices, although most acknowledge their professional relevance [8–11]. In Australia (2023), students demonstrated limited knowledge of environmentally sustainable pharmacy practice (ESPP), with few reporting curricular exposure, highlighting the need to develop ESPP content within pharmacy degrees [8]. In the USA (2024), pharmacy students generally rated medicines as more important than environmental concerns, despite awareness of their harmful effects, suggesting a tension between professional priorities and environmental responsibility [9]. In Canada (2025), students expressed strong interest in learning about the environmental impact of medicines, yet reported little curricular exposure, with calls for greater incorporation of sustainability and climate change content into the pharmacy curriculum [10].

Within the UK, momentum is building to embed sustainability into professional training. The UK-based Sustainability in Pharmacy Education Group has produced a framework aligning environmental sustainability with the General Pharmaceutical Council’s (GPhC) learning outcomes, endorsed by the Pharmacy Schools Council [12, 13]. Similarly, higher education regulators, including the Council of Deans of Health, have issued guidance for integrating sustainable healthcare across allied health curricula, underscoring the health–climate nexus [14].

In Northern Ireland, little is known about how pharmacy students conceptualise their role in environmental stewardship. The region’s community pharmacies dispensed nearly 45 million medicines in 2022–2023 [15], a figure expected to rise with population ageing and increasing multimorbidity. Such prescribing trends amplify the environmental consequences of pharmaceutical packaging, disposal, and supply chains. The Royal Pharmaceutical Society’s Green Pharmacy Guides underline both the professional commitment to adopt greener practices and the barriers pharmacists face, including resource constraints and competing priorities [16]. These developments highlight pharmacy’s central role in advancing sustainable healthcare. Yet, to achieve meaningful change, pharmacy education must equip graduates with the knowledge, confidence, and agency to act as environmental stewards within practice.

### Aim

To explore pharmacy students’ perceptions of environmental sustainability within their education and future professional roles, focusing on their awareness, attitudes, and perceived barriers or enablers to integrating sustainability in pharmacy education and practice.

## Method

This study was reported in line with the Consolidated Criteria for Reporting Qualitative Research (COREQ) 32-item checklist [17]. The checklist covers three domains: (1) research team and reflexivity, (2) study design, and (3) analysis and findings. Key elements included researcher credentials and reflexivity, purposive sampling across all years of the pharmacy programme, audio-recorded and verbatim-transcribed interviews, and inductive thematic analysis following Braun and Clarke’s six-phase approach [18]. Trustworthiness was ensured through reflexive journaling, peer debriefing, and maintaining an audit trail. Participant quotations are presented to illustrate themes, ensuring transparency between data and interpretation.

### Study design

An exploratory qualitative design was used, employing inductive thematic analysis as described by Braun and Clarke [18]. This approach allows themes to emerge from participants’ accounts without being constrained by pre-existing frameworks, making it well-suited to an under-researched area such as pharmacy students’ perspectives on environmental sustainability.

### Research setting

The study took place at a university in Northern Ireland offering an accredited four-year Master of Pharmacy (MPharm) programme. The programme is delivered primarily through face-to-face lectures, small-group workshops, and laboratory sessions, complemented by online learning resources and practice-based placements. This delivery model exposes students to both in-person and digital learning environments, which may influence engagement with contemporary issues such as environmental sustainability.

### Participant recruitment

A purposive, criterion-based sampling strategy guided by the principle of information power [19] was employed to ensure adequate variation across year of study and gender. Inclusion criteria were current enrolment in the four-year Master of Pharmacy (MPharm) programme at the university and willingness to participate in an individual interview. Exclusion criteria included students on leave of absence or those not enrolled in the MPharm programme. All eligible students were initially invited to express interest via university email announcements and short in-class announcements. From those who volunteered, participants were purposively selected to ensure representation across all four-year groups and an equal balance of male and female participants. Recruitment and interviews took place between January and May 2025, continuing until data saturation was achieved. Participation was voluntary, and no incentives were offered.

### Data collection

Semi-structured interviews were conducted between January and May 2025. All interviews were held online via Microsoft Teams for participant convenience and to ensure consistency. The interview guide (Supplementary File 1) was informed by literature on sustainability in healthcare and pharmacy education [8–10]. It included twelve open-ended questions exploring students’ awareness, experiences, and perceived barriers or enablers to environmental sustainability in pharmacy education and practice. The guide was reviewed by the research team and piloted with two students to ensure clarity and flow; pilot data were excluded from analysis.

All interviews were conducted by the second author (AM), a final-year Master of Pharmacy (MPharm) student trained in qualitative research methods under the supervision of an experienced pharmacy education researcher, the first author (KR). Conducting peer-to-peer interviews was considered advantageous in fostering openness and relatability among participants. However, to minimise potential response bias, participants were reminded that the interviewer was not involved in their teaching or assessment, that participation was voluntary, and that their responses would remain confidential. All interviews were audio-recorded with consent and transcribed verbatim.

### Data analysis

Data were analysed following Braun and Clarke’s six-phase process: (1) familiarisation, (2) coding, (3) theme development, (4) review, (5) definition, and (6) reporting [18]. NVivo 14 software supported coding and data management. Two researchers independently coded transcripts before meeting to resolve discrepancies and agree on themes. Analysis was inductive, grounded in participant accounts rather than pre-existing theory.

### Trustworthiness and rigour

Lincoln and Guba’s four criteria guided rigour [20]. Credibility was enhanced through multiple coders and regular team discussions; dependability by maintaining an audit trail; confirmability by reflexive discussions to minimise bias; and transferability by providing detailed contextual and demographic information.

### Reflexivity

The interviewer was a final-year Master of Pharmacy (MPharm) student trained in qualitative research methods, supervised by an experienced pharmacy education researcher. Their position as a peer may have encouraged open discussion, as participants could relate to shared experiences within the programme. However, this proximity also carried the potential for bias in both data collection and interpretation. To address this, the interviewer maintained a reflexive journal throughout the research process, documenting assumptions, preconceptions, and reflections after each interview. Regular supervisory meetings were held to discuss emerging themes and ensure that interpretation remained grounded in participants’ accounts rather than the researcher’s own views.

### Ethics approval

This study was performed in line with the principles of the Declaration of Helsinki. Approval was granted by the School of Biomedical Sciences Ethics Filter Committee (FCBMS) of Ulster University (Date: 21–03-2025 /No: FCBMS-24-144-A).

## Results

**Fig. 1:**
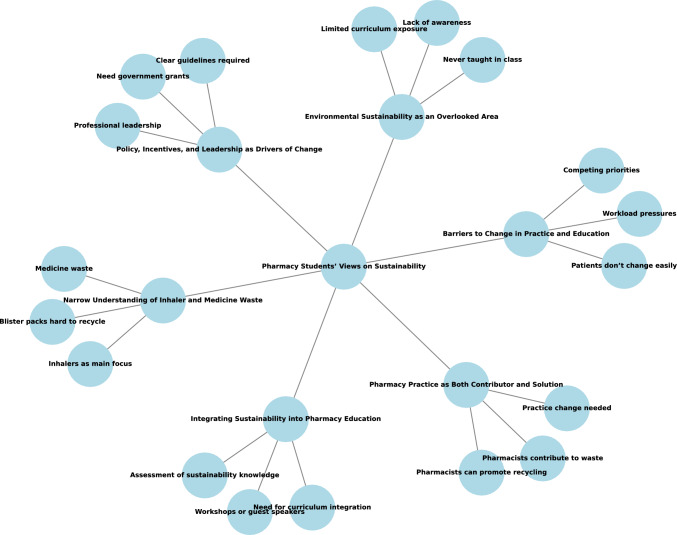
Visual mapping to explore relationships between codes and related concepts

Six themes were identified from sixteen interviews, each lasting between 30 and 60 min (mean = 45 ± 10 min), with data saturation reached at the 14th interview, when no new codes or themes were emerging. Saturation was confirmed through researcher consensus and by reviewing coded transcripts. Thematic analysis of the interview data generated six overarching themes: (1) Environmental sustainability as an overlooked area, (2) Narrow understanding of inhaler and medicine waste, (3) Pharmacy practice as both contributor and solution, (4) Integrating sustainability into pharmacy education, (5) Barriers to change in practice and education, and (6) Policy, incentives, and leadership as drivers of change. Visual mapping techniques were used to explore relationships between codes and categories (Fig. 1).

### Participant characteristics

The demographic details of the participants are shown in Table 1. A total of 16 pharmacy students participated in the study. Gender distribution was balanced, with 8 females and 8 males. Participants were purposively sampled across all four years of the MPharm programme, with equal representation from each year group (n = 4). Within each year, there was a balance of gender, with at least two male and two female participants represented. This ensured that perspectives from both genders were captured across the different stages of pharmacy education.

**Table 1:** Demographics of participants

Demographic characteristics	Frequency (n = 16)
Gender	
Female	8
Male	8
Year of study	
Year 1	4
Year 2	4
Year 3	4
Year 4	4

The six themes and their associated key codes are shown in Table 2.

**Table 2:** Themes and codes

Theme	Codes
Environmental sustainability as an overlooked area	Never taught in class Limited curriculum exposure Lack of awareness
Narrow understanding of inhaler and medicine waste	Inhalers as main focus Blister packs hard to recycle Medicine waste
Pharmacy practice as both contributor and solution	Pharmacists contribute to waste Pharmacists can promote recycling Practice change needed
Integrating sustainability into pharmacy education	Workshops or guest speakers Need for curriculum integration Assessment of sustainability knowledge
Barriers to change in practice and education	Patients don’t change easily Workload pressures Competing priorities
Policy, incentives, and leadership as drivers of change	Need government grants Clear guidelines required Professional leadership

1. Environmental sustainability as an overlooked area

Across all year groups, students consistently reported a lack of structured teaching on environmental sustainability within the pharmacy curriculum. They described sustainability as rarely given dedicated attention, often overlooked in both lectures and assessments.

One student expressed this clearly:

*“Not that I can remember.”* (Year 1 Female 1).

Another echoed this sense of absence, emphasising how unmemorable sustainability content had been:

*“Nothing is ringing a bell, so I'm gonna have to say no.”* (Year 2 Female 2).

Some students recalled only brief or superficial mentions of sustainability, embedded within wider lecture materials:

*“Maybe bits and pieces… one or two slides in a 30 slide PowerPoint… never getting the full attention that it deserves.”* (Year 3 Male 2).

A final year student reinforced this perception, noting that even when it was addressed, it lacked emphasis or assessment:

*“If we have, it’s been small or not examined on… I don’t really think that we have.”* (Year 4 Female 1).

2. Narrow understanding of inhaler and medicine waste

When discussing the environmental impact of pharmacy, students most frequently referred to inhalers. This reflects broader discourse in healthcare around their carbon footprint and the push to reduce the use of metered-dose inhalers (MDIs).

As one student explained:

*“Changing from MDI before… they are pushing for it not to be prescribed because of its effect on the environment.”* (Year 3 Male 2).

Students also drew attention to packaging waste, particularly the shift from bottles to blister packs. This was described as an everyday example of increasing and unrecyclable plastic use:

*“All tablets used to be in big bottles and decanted, now blister packs… that’s so much more plastic and you can’t recycle the foil.”* (Year 2 Female 2).

However, awareness of more complex issues, such as pharmaceutical manufacturing or excretion into the environment, was limited. A few participants did demonstrate awareness of device differences within inhalers:

*“Some inhalers are better for the environment than others… you can reduce wastage by choosing the right ones.”* (Year 4 Male 1).

Others framed sustainability more broadly as waste minimisation within pharmacy practice, focusing on reducing unnecessary packaging or prescriptions:

*“In practice… it’s about not over excessive packaging and reducing waste.”* (Year 2 Male 1).

3. Pharmacy practice as both contributor and solution

Students described pharmacy as a setting that generates significant waste, but also recognised its potential to contribute positively to environmental sustainability. They identified practical examples where sustainable practices were already emerging.

Digitalisation was one area highlighted as a positive shift, with students noting reduced reliance on paper due to electronic systems:

*“There seem to be a lot less paper usage and everything was online… patients’ files were online.”* (Year 2 Female 1).

Medicine returns and proper disposal were also seen as central to reducing environmental harm. One participant explained how this was embedded in practice:

*“We’d always tell patients to bring medicines back… we’d dispose of them properly instead of the regular waste.”* (Year 3 Male 2).

Another reinforced this view, stressing the environmental value of structured return schemes:

*“Patient returns… like bringing back medicines, that’s really good for the environment because then you can dispose of them in the right way.”* (Year 4 Female 2).

Innovations within hospital pharmacy settings were also mentioned, with students observing initiatives aimed at reducing plastic usage:

*“In hospital, I saw a green team encouraging reusable bags for transporting medications instead of plastic.”* (Year 1 Female 1).

4. Integrating sustainability into pharmacy education

Participants strongly advocated for embedding sustainability into the pharmacy curriculum, stressing that it should receive greater visibility and relevance. They also favoured varied and interactive approaches rather than relying solely on lectures.

Some students highlighted the value of guest speakers who could bring real-world experience into the classroom:

*“Maybe if there was a speaker… showing what it looks like in practice rather than just a lecture.”* (Year 2 Female 1).

Experiential opportunities were also seen as particularly impactful. For example, one participant suggested visits to sustainability-focused organisations to see practical applications firsthand:

*“External speakers would be the best… even a day trip to a sustainability company, so you can actually see the impact.”* (Year 4 Female 2).

Others recommended making sustainability teaching more interactive and engaging, integrated into assessments and class activities:

*“Lectures, assignments, even quick quizzes… students would be very open to that.”* (Year 3 Male 2).

One student neatly captured the consensus by reiterating the value of real-world examples shared by external experts:

*“Probably best delivered through guest speakers with real experience in sustainability.”* (Year 1 Female 1).

5. Barriers to change in practice and education

Although students recognised the importance of sustainability, they also identified significant barriers that could hinder its adoption in both education and professional practice.

Patient resistance to change was one concern, with some students highlighting generational differences:

*“Older patients don’t like change either.”* (Year 2 Female 2).

Professional habits and behavioural inertia were also described as barriers, with students noting the difficulty of shifting established routines:

*“It could slow things down… people are so used to what they’re doing, it’s hard to change overnight.”* (Year 4 Female 2).

Others pointed to structural pressures within the pharmacy curriculum and practice settings, where sustainability may struggle to compete with other priorities:

*“There’s already so much going on… but if they’re adding new things, I don’t see why they couldn’t add this.”* (Year 3 Female 1).

Finally, variability across the profession was noted, with awareness and engagement perceived as inconsistent among pharmacists:

*“Pharmacists are different… some are very aware, others not at all.”* (Year 2 Male 2).

6. Policy, incentives, and leadership as drivers of change

Students emphasised that sustainability in pharmacy would only be successful if supported by systemic drivers such as policy, leadership, and financial incentives. They recognised that individual goodwill alone would not be sufficient to drive meaningful change.

Some students highlighted the importance of leadership at both professional and organisational levels:

*“Whether it comes from the pharmacist or people above them… something can be done, but it’s a big sector.”* (Year 2 Female 2).

Others pointed out that adequate resources would be essential to support sustainability initiatives in practice:

*“If the resources were given to them, they’d have no issues.”* (Year 2 Male 1).

Financial incentives were repeatedly framed as powerful levers for change, with grants or rewards seen as a way to encourage uptake:

*“Probably grants would have to be put in place… if you can show you reduced waste, then you could receive a grant.”* (Year 4 Female 2).

One student summarised this pragmatic perspective, arguing that incentives could help overcome resistance to change:

*“Nobody likes change, but if you put a bit of money behind it, we’d all be for it.”* (Year 3 Male 2).

## Discussion

This study explored pharmacy students’ perceptions of environmental sustainability in education and practice. Six themes were identified, highlighting limited curricular exposure, a narrow understanding of environmental impact, recognition of both challenges and opportunities in practice, and the need for systemic drivers of change.

This study found that pharmacy students across all four years viewed environmental sustainability as largely overlooked within their curriculum. While this study included participants from all four years of the MPharm programme, there were no marked differences in responses between year levels. Students across all cohorts consistently reported limited exposure to environmental sustainability within the curriculum. Although it might be expected that senior students would have encountered greater coverage through lectures, placements, or project work, this was not evident in their accounts. This consistency across year groups reinforces the finding that environmental sustainability is not yet systematically embedded or scaffolded throughout the programme. It also highlights the need for a longitudinal and progressive integration of sustainability concepts, introducing foundational awareness in early years and developing applied, practice-based competencies in later years, to ensure coherent learning across the MPharm trajectory. Some recalled occasional mentions, but these were minimal, fragmented, or hidden within broader topics. This supports international evidence showing that sustainability is rarely embedded in pharmacy teaching in a consistent way [8, 9]. Students noted that the absence of sustainability in assessments further limits its recognition as a professional competency. This gap has serious implications, as pharmacists are key to reducing waste, supporting patients, and promoting sustainable healthcare [21]. Without structured training, graduates may feel unprepared to take on these responsibilities. The mismatch between urgent climate goals and limited teaching reflects what others call a “curriculum–practice gap” [22]. Fragmented coverage may also create narrow views of sustainability, focusing only on “bits and pieces” rather than wider issues like carbon emissions and pharmaceutical waste. Students’ voices highlight demand for stronger curriculum inclusion [8]. Addressing this gap requires embedding sustainability throughout pharmacy education to ensure future practitioners are prepared to meet both professional and planetary health responsibilities.

Students’ emphasis on inhalers and packaging reflects dominant sustainability discourses in UK healthcare, where visible consumer-level issues often overshadow upstream production and supply-chain impacts. The prominence of inhalers reflects UK debates, where MDIs are linked to high carbon footprints due to hydrofluorocarbon propellants [23, 24]. Students recognised the shift toward lower-impact DPIs, suggesting their awareness is shaped more by media and policy than by curriculum. Knowledge of wider issues, such as API production, pharmaceutical waste in water, or supply chain emissions, was rarely mentioned, echoing global findings that students equate sustainability with waste and recycling [8, 9]. Packaging, especially blister packs, was another concern, with students noting recycling challenges [25]. This focus on packaging reflects an end-user perspective, what is visible to consumers, rather than an awareness of upstream pharmaceutical supply chain emissions or manufacturing waste. Researches show that while packaging accounts for only a fraction of the total environmental burden of pharmaceuticals, production and transportation contribute far greater impacts [26, 27]. Without explicit curricular coverage of these upstream processes, students may overemphasise visible waste at the expense of understanding systemic sustainability challenges. Some recognised differences between inhaler types and waste minimisation, linking sustainability to pharmacists’ roles in prescribing and medicines optimisation [28, 29]. Overall, student awareness was limited, requiring broader education on pharmacy’s full environmental impact.

Students recognised that pharmacy produces waste but also has the potential to support sustainability. Students identified visible sustainability practices such as digitalisation and medicines return schemes, reflecting awareness shaped by tangible, operational aspects of pharmacy practice. However, this practical focus reveals a limited systems perspective, as students tended to equate sustainability with waste reduction and recycling rather than upstream interventions such as sustainable procurement, prescribing optimisation, or green manufacturing. Digitalisation was viewed positively, with reduced paper use seen as progress, consistent with findings that digital technologies can cut material waste, though energy use remains a concern [30]. Medicines returns were valued for preventing pharmaceuticals entering general waste, echoing wider evidence of the pharmacist’s role in safe disposal and counselling [28]. Students also recalled hospital initiatives, such as reusable transport bags, reflecting NHS efforts to embed “green champions” and reduce plastics [31]. These examples show how experiential exposure, such as placements, shapes understanding. However, students’ focus was mainly on visible waste reduction, overlooking broader impacts such as manufacturing, transport, and prescribing [32–34]. This highlights the need for education to expand awareness beyond surface-level sustainability.

Students strongly supported embedding environmental sustainability into the pharmacy curriculum, stressing the need for greater visibility and relevance. They favoured diverse and interactive approaches over lectures, reflecting wider evidence that effective sustainability education requires active, context-specific learning [35, 36]. Guest speakers, visits to sustainability-focused organisations, and real-world case examples were suggested, aligning with experiential pedagogies shown to foster environmental responsibility [37]. Interactive strategies such as assignments, quizzes, and case discussions were also highlighted, consistent with research showing active learning improves knowledge retention and motivation [38]. Students argued sustainability should be a core element of professional identity, not a peripheral topic, echoing findings where learners expressed frustration at its curricular absence [9, 39]. Embedding sustainability longitudinally across programmes would ensure consistent exposure [1, 40]. Their views align with frameworks such as FIP’s Sustainability Development Goals, which call for sustainability as a key competency for future pharmacists [41].

While students valued integrating sustainability into pharmacy, they identified barriers that could hinder progress. Key challenges included resistance from patients and professionals, behavioural inertia, curriculum pressures, and uneven awareness across the profession. Patient resistance was commonly noted, particularly among older individuals reluctant to change, reflecting wider evidence that familiarity with established practices can impede greener alternatives [42]. Professional habits and competing priorities were also seen as obstacles, consistent with implementation science findings that inertia and limited incentives slow adoption [43]. Structural pressures within the curriculum were highlighted, with students concerned that an already full timetable may lead to sustainability being deprioritised. This mirrors international reports of difficulties introducing new topics without displacing existing ones [44]. However, students believed these barriers could be addressed by embedding sustainability into existing modules and activities, ensuring integration without overburdening the curriculum.

Students emphasised that embedding sustainability in pharmacy requires systemic drivers such as policy, leadership, and financial incentives. While individual behaviour change was seen as valuable, participants recognised that meaningful progress depends on institutional and policy-level commitment. This aligns with wider evidence that sustainability leadership in healthcare and higher education often relies on motivated individuals rather than coordinated strategy, leading to fragmented implementation [45, 46]. In pharmacy education, similar challenges arise when sustainability lacks explicit inclusion in accreditation standards or regulatory expectations, resulting in variable curricular adoption across universities [47–49]. Competing institutional priorities, such as meeting professional competencies, maintaining accreditation, and managing limited curricular space, can further constrain integration. To address this, leadership from regulatory bodies and universities is essential to embed environmental sustainability within accreditation frameworks, programme specifications, and quality assurance processes [50]. Clear policy direction, such as that demonstrated by the NHS Net Zero Plan and the Royal Pharmaceutical Society’s Greener Pharmacy Framework, can serve as exemplars for aligning educational goals with national sustainability strategies [12, 16, 31]. Embedding accountability and incentives at both educational and practice levels would help shift sustainability from a voluntary aspiration to a structural expectation within the profession.

It is important to recognise that environmental sustainability in pharmacy education is influenced by national priorities and policy contexts. Different countries are at varying stages of developing and implementing sustainability strategies within healthcare and higher education. Consequently, the findings of this study, while offering valuable insight into the Northern Ireland context, may not be directly transferable to regions where sustainability is already embedded more systematically in curricula, or conversely, where awareness remains limited. Nevertheless, the patterns observed here echo international studies highlighting similar educational gaps, suggesting broader relevance for pharmacy education reform globally.

### Limitations of the study

This study has several limitations that should be acknowledged. First, the research was conducted at a single university in Northern Ireland, which may limit the transferability of findings to pharmacy students in other regions or educational contexts. Differences in curricula, regulatory frameworks, and cultural perspectives on sustainability may lead to varied experiences elsewhere. Second, the reliance on self-reported views in interviews introduces the possibility of recall bias or social desirability bias, as participants may have under- or overstated their awareness of sustainability issues. Finally, as data collection relied on students’ perceptions, the study cannot establish how sustainability is formally embedded in the curriculum but rather reflects how it is received and understood by learners.

### Implications for policy, practice, and research

Findings emphasise the need to integrate environmental sustainability into pharmacy curricula through interactive and experiential approaches, aligned with FIP Workforce Development Goals. Embedding such content feasibly and sustainably requires thoughtful curriculum design. Using a constructively aligned approach, sustainability-related learning outcomes can be explicitly linked to teaching and assessment strategies, ensuring consistency and relevance. A spiral curriculum model allows sustainability principles to be revisited across different years of study, building competence and confidence over time. Embedding environmental sustainability as a cross-cutting theme, rather than a stand-alone topic, aligns with guidance from the General Pharmaceutical Council and global Education for Sustainable Development frameworks. This approach supports progressive learning, authenticity, and integration within existing accreditation and professional standards, making curriculum reform achievable and durable. Future research should explore sustainability education across schools, track changes in student attitudes, assess experiential strategies, and investigate how policy, incentives, and cross-disciplinary collaboration enable sustainable practices in pharmacy.

## Conclusion

This study highlights a significant gap between pharmacy students’ recognition of environmental sustainability as a professional responsibility and its limited integration within current curricula. Students called for interactive, practice-based learning to strengthen their competence and confidence in sustainable pharmacy practice. Addressing this requires coordinated action across educational, professional, and policy levels, embedding sustainability into pharmacy curricula, aligning institutional priorities with national environmental goals, and providing leadership and incentives to enable change. By equipping future pharmacists with the skills and motivation to act as environmental stewards, pharmacy education can play a pivotal role in advancing sustainable healthcare and mitigating pharmacy’s contribution to climate change.

## Supplementary Information

Below is the link to the electronic supplementary material.

Supplementary file1 (DOCX 30 KB)

## Data Availability

The datasets generated during and/or analysed during the current study are available from the corresponding author on reasonable request.
