# Supplementary material for: Future pharmacists and climate action: a qualitative study of students’ views on environmental sustainability in education and practice
Source: Int J Clin Pharm. 2025 Nov 22;48(2):656–66. doi: 10.1007/s11096-025-02053-4 (PMC12992404; doi:10.1007/s11096-025-02053-4)
Supplement: Supplementary file 1 — Supplementary file1 (DOCX 30 KB) [file 11096_2025_2053_MOESM1_ESM.docx]

**Interview Questions (In-depth interview)**

**Understanding Perspectives on Environmental Sustainability**

- From your perspective, what does environmental sustainability mean in the context of pharmacy education and practice?
- How do you believe environmental sustainability relates to the role of pharmacists in healthcare?

**Awareness and Engagement**

- How aware do you feel pharmacy students are about environmental sustainability issues within the pharmaceutical field?
- Have you personally been involved in any activities or initiatives related to environmental sustainability during your pharmacy education?

**Challenges and Barriers**

- In your opinion, what are some of the main challenges hindering the integration of environmental sustainability principles into pharmacy education and practice?
- Have you encountered any specific barriers or obstacles in trying to promote environmental sustainability within your pharmacy studies or practice?

**Opportunities and Solutions**

- What opportunities do you see for pharmacy education programs to better incorporate environmental sustainability into their curricula?
- Can you suggest any innovative approaches or solutions that could help promote environmental sustainability in pharmacy practice?

**Reflection and Future Outlook**

- Looking ahead, how do you envision the role of pharmacists evolving in addressing environmental sustainability concerns within the pharmaceutical industry?
- What changes would you like to see in pharmacy education and practice to further emphasize environmental sustainability?

**Closing**

- Is there anything else you would like to share or discuss regarding environmental sustainability in pharmacy education and practice?
- Thank you for your time and participation. Would you be open to future follow-up discussions if needed?
